# Draft Genome Sequence of *Kozakia baliensis* SR-745, the First Sequenced *Kozakia* Strain from the Family *Acetobacteraceae*

**DOI:** 10.1128/genomeA.00594-14

**Published:** 2014-06-26

**Authors:** Jochen Schmid, Steven Koenig, André Pick, Fabian Steffler, Shosuke Yoshida, Kenji Miyamoto, Volker Sieber

**Affiliations:** a Chemistry of Biogenic Resources, Technische Universität München, Straubing, Germany; b Department of Biosciences and Informatics, Keio University, Yokohama, Kanagawa, Japan

## Abstract

*Kozakia baliensis* belongs to the family *Acetobacteraceae* and was described for the first time in 2002. These acetic acid bacteria are able to produce acetic acid from various carbon sources and 2- and 5-keto-d-gluconate from glucose. The novel *K. baliensis* strain SR-745 was isolated from a pineapple fruit bought in a German supermarket. The strain produces large amounts of organic acids when grown on glucose-containing medium and accepts also glycerol, fructose, mannitol, and sucrose as a C source. When grown under light and high-oxygen conditions in submerged culture, the production of a pink pigment is observed after 72 h.

## GENOME ANNOUNCEMENT

*Kozakia baliensis* SR-745 was obtained by classical microbiological isolation techniques from a pineapple bought in a German supermarket. The strain was identified as a *Kozakia* strain by 16S rRNA analysis. A BLAST analysis indicated that *K. baliensis* NBRC 16679 (100% identity in 16S rRNA) is its closest neighbor (1). The whole-genome shotgun sequence of *K. baliensis* SR-745 was obtained by one Illumina MiSeq run and one Illumina GAIIx run independently performed in Germany and Japan, respectively, based on the same DNA sample. The genomic DNA of *K. baliensis* SR-745 was obtained using an adapted method of Chen and Kuo (2), and shearing and library preparation were done in accordance with the Illumina TruSeq DNA sample preparation guide version 2 (3); the only noteworthy deviation is the substitution of the step “purify ligation products (gel method only)” with “purify cDNA construct” from the TruSeq small RNA sample preparation guide (4) for the sequencing in Germany.

The sequencing in Germany yielded 1,211,830 paired-end reads, with read lengths ranging from 35 bp to 151 bp (median, 150 bp). The sequencing in Japan yielded 26,561,095 single reads, with an average length of 109 bp. The reads from Germany were trimmed and quality filtered using TrimmingReads.pl (5), cutadapt (6), and DynamicTrim.pl (7). Quality assessment was done using FastQC (http://www.bioinformatics.babraham.ac.uk/projects/fastqc/) and SolexaQA (7). After processing, 759,574 forward and 919,579 reverse reads remained, with 1,145,218 paired reads and 533,935 singletons. The Japanese reads were trimmed and quality filtered using CLC Genomics Workbench version 6.5.1, and subsequently, TrimmingReads.pl and DynamicTrim.pl. After processing, 25,849,107 reads remained. Assemblies of the combined data from Germany and Japan were carried out using Velvet 1.2.08 (8). All *k*-mer values from 15 to 83 were examined. The assembly using a *k*-mer length of 55 yielded the highest total sequence length (3,172,521 bp; *N*_50_, 92,060 bp, without the PhiX contig) and was used for further analyses. The G+C content of the assembled contigs is 57.5%. The draft genome is made up of 106 scaffolds, which are composed of 114 contigs. Annotation was carried out by uploading the generated scaffolds to RAST (9), which found 3,151 coding sequences comprising 1,390 genes in several subsystems. A total number of 48 RNA genes were detected by RAST analysis. Fifty-five genes were identified in the monosaccharide subsystem, including those for the utilization of ribose and xylose and the metabolism of mannose, galactose, and gluconate and ketogluconate.

### Nucleotide sequence accession numbers.

This whole-genome shotgun project has been deposited at DDBJ/EMBL/GenBank under the accession no. JNAB00000000. The version described in this paper is version JNAB01000000.

## References

[1] Zhang Z Schwartz S Wagner L Miller W 2000 A greedy algorithm for aligning DNA sequences. J. Comput. Biol. 7:203–214. 10.1089/10665270050081478 10890397

[2] Chen WP Kuo TT 1993 A simple and rapid method for the preparation of gram-negative bacterial genomic DNA. Nucleic Acids Res. 21:2260. 10.1093/nar/21.9.2260 8502576 PMC309503

[3] Illumina 2011 TruSeq DNA sample preparation v2 guide. Illumina, San Diego, CA. http://supportres.illumina.com/documents/myillumina/f5f619d3-2c4c-489b-80a3-e0414baa4e89/truseq_dna_sampleprep_guide_15026486_c.pdf

[4] Illumina 2011 TruSeq small RNA sample preparation guide. Illumina, San Diego, CA. http://supportres.illumina.com/documents/documentation/chemistry_documentation/samplepreps_truseq/truseqsmallrna/truseq-small-rna-sample-prep-guide-15004197-f.pdf

[5] Patel RK Jain M 2012 NGS QC Toolkit: a toolkit for quality control of next generation sequencing data. PLoS One 7:e30619. 10.1371/journal.pone.0030619 22312429 PMC3270013

[6] Martin M 2011 Cutadapt removes adapter sequences from high-throughput sequencing reads. EMBnet.journal 17:10–12. 10.14806/ej.17.1.200

[7] Cox MP Peterson DA Biggs PJ 2010 SolexaQA: at-a-glance quality assessment of Illumina second-generation sequencing data. BMC Bioinformatics 11:485. 10.1186/1471-2105-11-485 20875133 PMC2956736

[8] Zerbino DR Birney E 2008 Velvet: algorithms for *de novo* short read assembly using de Bruijn graphs. Genome Res. 18:821–829. 10.1101/gr.074492.107 18349386 PMC2336801

[9] Overbeek R Begley T Butler RM Choudhuri JV Chuang HY Cohoon M de Crécy-Lagard V Diaz N Disz T Edwards R Fonstein M Frank ED Gerdes S Glass EM Goesmann A Hanson A Iwata-Reuyl D Jensen R Jamshidi N Krause L Kubal M Larsen N Linke B McHardy AC Meyer F Neuweger H Olsen G Olson R Osterman A Portnoy V Pusch GD Rodionov DA Rückert C Steiner J Stevens R Thiele I Vassieva O Ye Y Zagnitko O Vonstein V 2005 The subsystems approach to genome annotation and its use in the project to annotate 1000 genomes. Nucleic Acids Res. 33:5691–5702. 10.1093/nar/gki866 16214803 PMC1251668

